# MLKL-Driven Inflammasome Activation and Caspase-8 Mediate Inflammatory Cell Death in Influenza A Virus Infection

**DOI:** 10.1128/mbio.00110-23

**Published:** 2023-02-28

**Authors:** Xuqiu Lei, Yongzhi Chen, Egil Lien, Katherine A. Fitzgerald

**Affiliations:** a Program in Innate Immunity, Department of Medicine, University of Massachusetts Chan Medical School, Worcester, Massachusetts, USA; b Centre of Molecular Inflammation Research, Department of Clinical and Molecular Medicine, Norwegian University of Science and Technology (NTNU), Trondheim, Norway; Stony Brook University

**Keywords:** cell death, inflammasome, MLKL, caspases, influenza virus

## Abstract

Influenza A virus (IAV) triggers multiple programmed cell death pathways, including MLKL-dependent necroptosis, caspase-8-dependent apoptosis, and caspase-1-dependent pyroptosis in myeloid cells. All three pathways share common upstream regulators, namely, ZBP1 and RIPK3. Yet, the molecular mechanism underlying IAV-induced inflammasome activation remains unclear. Here, we demonstrate that MLKL promotes inflammasome activation and IL-1β processing in IAV-infected macrophages. MLKL drives NLRP3 inflammasome activation through potassium efflux. In the absence of the MLKL-inflammasome axis, caspase-8 coordinates the maturation and secretion of IL-1β. MLKL alone is dispensable for host inflammatory responses to IAV in vivo. Taken together, MLKL and caspase-8 serve as redundant mechanisms by which to drive an inflammatory form of cell death in response to an IAV infection.

## INTRODUCTION

Influenza A viruses (IAV) are negative-stranded RNA viruses that can cause severe inflammation and tissue damage in the lung. An IAV infection triggers multiple programmed cell death pathways, including apoptosis, necroptosis, and pyroptosis, all of which require the master regulators ZBP1 and RIPK3 (1, 2). ZBP1 senses the double-stranded helical z-RNA that is synthesized during viral replication (3) and engages RIPK3 to activate MLKL-dependent necroptosis and FADD/caspase-8-driven apoptosis. RIPK3 phosphorylates MLKL, leading to MLKL membrane translocation, pore formation, and, eventually, membrane rupture (4, 5). RIPK3 can also complex with RIPK1, FADD, and caspase-8, which induces caspase-8 autocleavage that is independent of RIPK1 and RIPK3 kinase activity (1, 2). Active caspase-8 further cleaves cellular proteins, including caspase-3 and caspase-7, to drive apoptosis.

Pyroptosis is driven by gasdermins that are activated by caspase-1 forming inflammasomes. IAV-induced inflammasome activation typically involves the assembly of a multiprotein oligomeric complex that is composed of the Nod-like receptor NLRP3, the adaptor ASC, and caspase-1 (6–9). Prior to inflammasome assembly, the expression of NLRP3 and precursors of the proinflammatory cytokines IL-1β and IL-18 are induced in a priming step, usually through innate signaling pathways, such as Toll-like receptors (10, 11). Inflammasome assembly then leads to caspase-1 activation, which cleaves GSDMD into an N-terminal fragment and IL-1β/18 into bioactive products (12–14). The GSDMD-N fragment forms pores on the plasma membrane, leading to pyroptosis, the secretion of IL-1β/18, and a plethora of other alarmins or danger signals. In contrast to MLKL and FADD/caspase-8, whether the inflammasome components directly interact with ZBP1-RIPK3 during an IAV infection is unclear. Although it has been proposed that the pyroptosis pathway acts in parallel with apoptosis and necroptosis or downstream of caspase-8, the molecular mechanisms underlying inflammasome activation during an IAV infection remain to be defined.

Emerging evidence suggests that apoptosis and necroptosis pathways can cross-talk to pyroptosis. Caspase-8 can directly cleave GSDMD and IL-1β to promote inflammatory pyroptotic cell death in fungal and bacterial infections (15–17). In these cases, GSDMD pores can cause potassium efflux and can activate the NLRP3 inflammasome, thereby contributing to the release of IL-1β (13, 15, 18). Caspase-3/7 downstream of caspase-8 in the apoptosis pathway can negatively regulate pyroptosis by cleaving and inactivating GSDMD (19). Active MLKL in the necroptosis pathway can also trigger the activation of the NLRP3 inflammasome via potassium efflux and can promote IL-1β secretion independently of GSDMD (20). Given that all three pathways are induced in IAV-infected cells and share upstream regulators, we investigated whether and how apoptosis and necroptosis pathways regulate the pyroptosis pathway.

## RESULTS

### MLKL promotes IAV-induced inflammasome activation.

Bone marrow-derived macrophages (BMDMs) express the key molecules that coordinate apoptosis, necroptosis, and pyroptosis. As a result, these cells are useful in the study of the interplay of cell death pathways during IAV infection. Although IAV itself provides a priming signal that is sufficient for inflammasome activation (21), we pretreated the BMDMs with the TLR1/2 agonist Pam3CSK4 to enhance the upregulation of pro-IL-1β, which allowed for a more robust detection of mature IL-1β (Fig. S1). This also mimics the condition of IAV and bacterial coinfection, such as Streptococcus pneumoniae (22).

10.1128/mbio.00110-23.1 FIG S1 Effects of Pam3CSK4 priming. (A–D) BMDMs were untreated or primed with Pam3 for 3 h. The cells were then stimulated with 10μM NG for 1 h, 200 μg/mL silica for 24 h, or PR8 at a MOI of 4 for 24 h. (A) Western blots showing GSDMD and GAPDH in the cell lysates. (B) Cell death was monitored hourly after PR8 infection. The IL-1β (C) and IFN-β (D) levels in the supernatant are shown. The data are presented as the mean with SD. The data were analyzed via a two-way ANOVA (C) or Welch’s *t* test (D). ns, not significant; *, *P* < 0.0332; **, *P* < 0.0021; ***, *P* < 0.0002; ****, *P* < 0.0001. Download FIG S1, PDF file, 1.0 MB. Copyright © 2023 Lei et al. 2023 Lei et al. https://creativecommons.org/licenses/by/4.0/ This content is distributed under the terms of the Creative Commons Attribution 4.0 International license.

**FIG 1:**
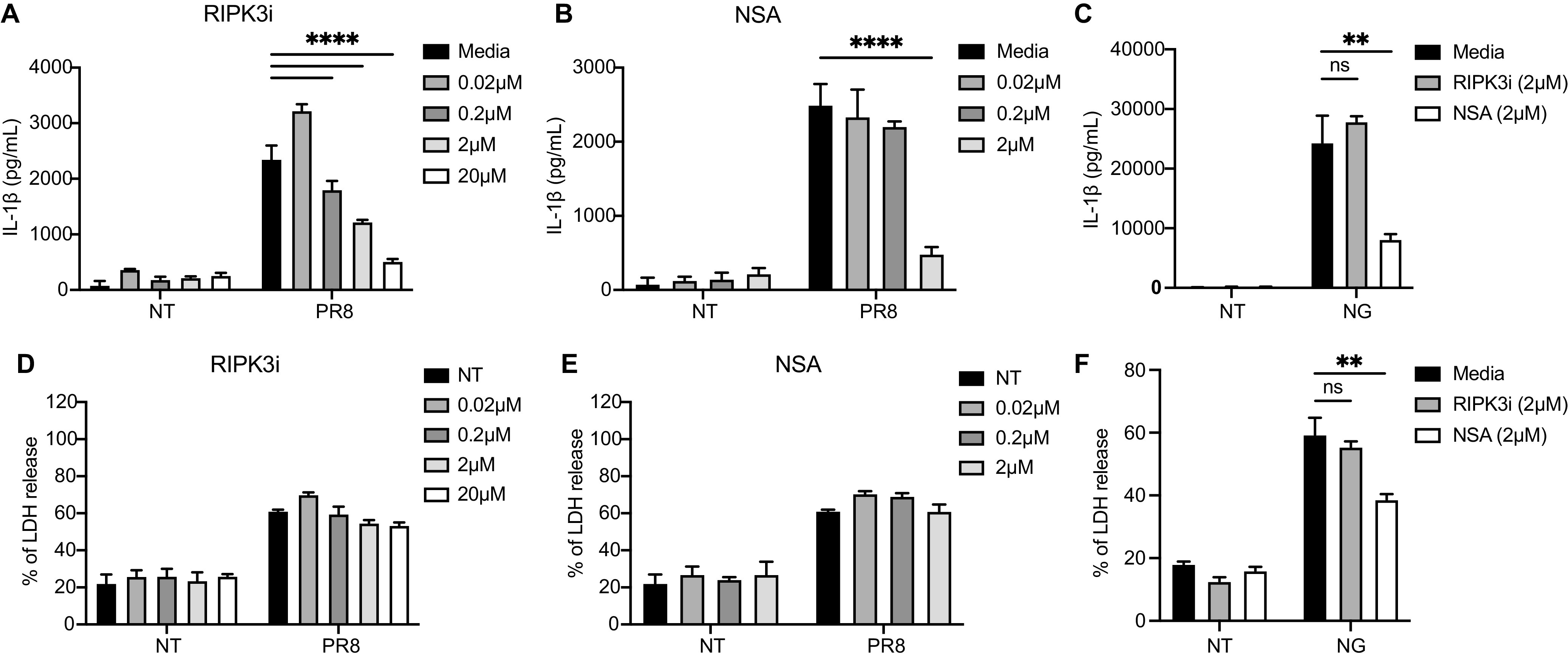
Necroptosis regulates IAV-induced IL-1β production but not cell death. (A, B, D, and E) Bone marrow-derived macrophages (BMDMs) were primed with 100 ng/mL Pam3CSK4 (Pam3) for 3 h and were infected with influenza A virus H1N1 strain PR8 at a multiplicity of infection (MOI) of 2 or with no virus (NT). The indicated concentrations of RIPK3 inhibitor (RIPK3i, GSK’872) and NSA were added 1 h later. The IL-1β (panels A and B) and LDH (panels D and E) levels in the supernatant were determined 18 h after infection. (C, F) BMDMs were primed with Pam3 for 3 h and incubated with inhibitors for 1 h. The cells were then stimulated with nigericin (NG). The IL-1β (panel C) and LDH (panel F) levels in the supernatant were determined 1 h later. The data show one representative experiment of three independent experiments and are presented as the mean with the SD. N = 3 to 4 (replicate wells). The data were analyzed via a two-way ANOVA. ns, not significant; *, *P* < 0.0332; **, *P* < 0.0021; ***, *P* < 0.0002; ****, *P* < 0.0001.

To investigate whether the necroptosis pathway is involved in IAV-induced inflammasome activation, we tested small molecules that block RIPK3 kinase activity (GSK’872) or RIPK3-MLKL interaction (Necrosulfonamide [NSA]) (4), and measured the release of IL-1β and lactate dehydrogenase (LDH) in the supernatant. RIPK3 inhibition and NSA blocked the release of IL-1β in a dose-dependent manner (Fig. 1A and B). However, neither affected IAV-induced cell death, as indicated by comparable LDH release (Fig. 1D and E). The potassium ionophore nigericin activates the canonical NLRP3 inflammasome, in which GSDMD is the primary cell death executor and is required for IL-1β secretion (13, 14). Canonical NLRP3 inflammasome-mediated cell death and the release of IL-1β were inhibited by NSA, which also targets GSDMD (23), but not by the RIPK3 inhibitor GSK’872 (Fig. 1C and F). These results suggest that active MLKL, possibly phosphorylated by RIPK3, is involved in IAV-induced inflammasome activation but not in canonical inflammasome activation.

**FIG 2:**
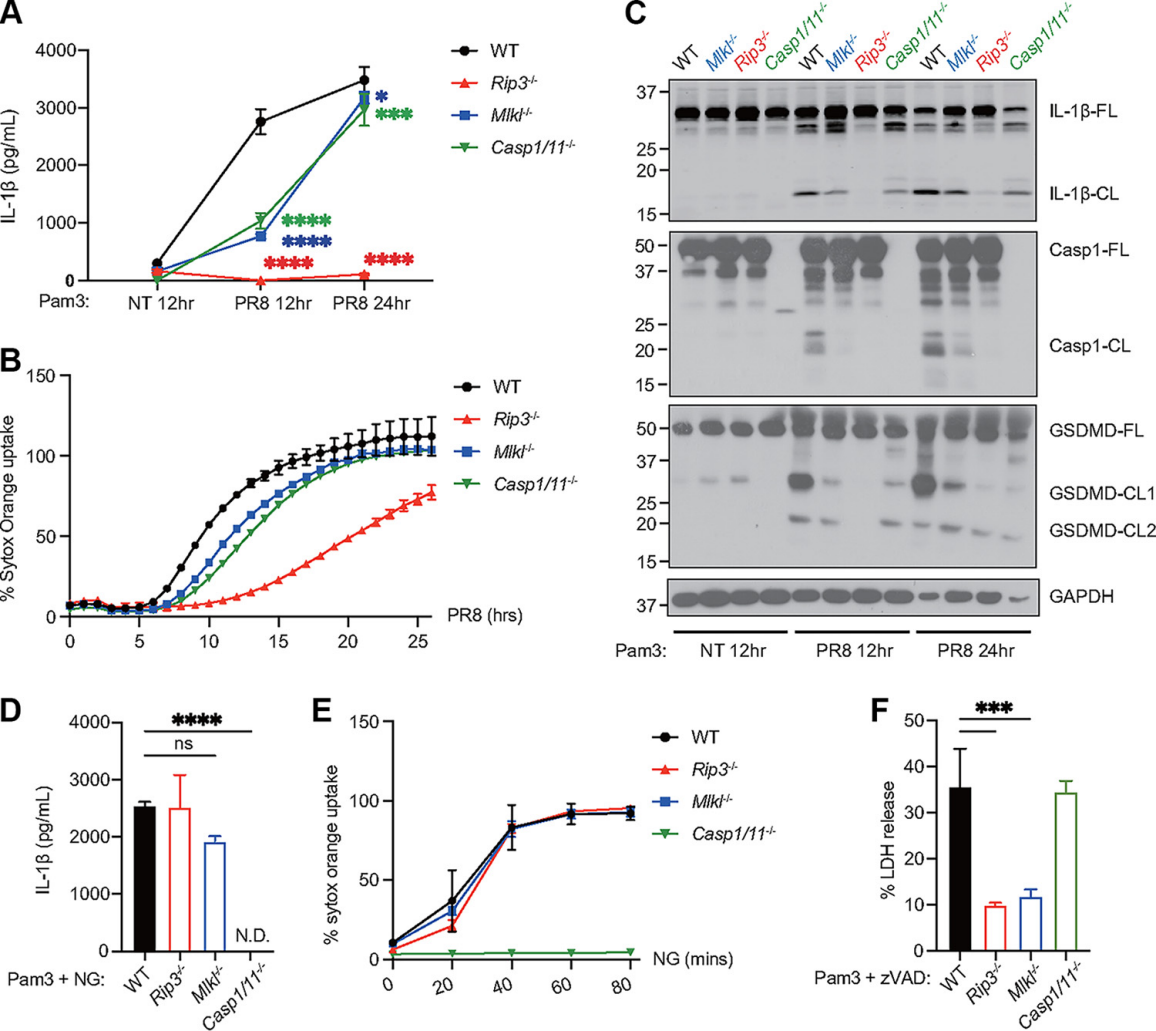
MLKL promotes inflammasome activation during IAV infection. (A) WT, *Rip3^−/−^*, *Mlkl^−/−^*, and *Casp1/11^−/−^* BMDMs were primed with Pam3 for 3 h. The cells were then infected with PR8 at a MOI of 2 or with no virus (NT). The IL-1β level in the supernatant was determined 12 and 24 h after infection. (B) To monitor cell death in real-time, BMDMs infected as in (panel A) were incubated with SYTOX Orange and Hoechst and were imaged hourly using a Cytation 5 system. The percentage of double-positive cells (membrane permeabilized cells) in the Hoechst-positive cells (total cell numbers) is shown. (C) Western blots detecting IL-1β and caspase-1 in the composite of cell lysate and supernatant as well as GSDMD and GAPDH in the cell lysate. The full-length (-FL) or cleaved form (-CL) of the protein is indicated. (D) WT, *Rip3^−/−^*, *Mlkl^−/−^*, and *Casp1/11^−/−^* BMDMs were primed with Pam3 for 3 h and were stimulated with nigericin (NG). The IL-1β level in the supernatant was determined 1 h later. N.D., not detected. (E) Cell death after the NG treatment was monitored as in (panel B) every 20 min for 80 min. (F) WT, *Rip3^−/−^*, *Mlkl^−/−^*, and *Casp1/11^−/−^* BMDMs were treated with Pam3 and Z-VAD-FMK (zVAD) for 24 h. Cell death was determined by measuring the release of LDH in the supernatant. The data show one representative experiment of at least two independent experiments and are presented as the mean with the SD. N = 3 (replicate wells). The data were analyzed via a two-way ANOVA. ns, not significant; *, *P* < 0.0332; **, *P* < 0.0021; ***, *P* < 0.0002; ****, *P* < 0.0001.

We compared WT cells and cells lacking RIPK3, MLKL, or caspase-1/11 for their responses to IAV infections (Fig. 2A and B). Real-time cell death was determined using automated imaging and was indicated by the percentage of SYTOX Orange permissive cells in the total cells (Fig. 2B). Consistent with the results of previous reports that show RIPK3 as the master upstream regulator (1, 2), we observed a significant delay of cell death and diminished IL-1β production from RIPK3 KO BMDMs (Fig. 2A and B). A deficiency of caspase-1/11 or MLKL led to a slight delay in cell death at the early stage of infection, but they were largely dispensable at later stages (Fig. 2B). As expected, caspase-1/11 DKO cells produced significantly less IL-1β, compared to the WT at 12 h postinfection (Fig. 2A). Consistent with the cells that were treated with MLKL inhibitors, MLKL KO cells had significantly less IL-1β release at this time point (Fig. 2A). The remaining level of IL-1β in MLKL KO cells was comparable to that of caspase-1/11 DKO cells. Moreover, both MLKL KO cells and caspase-1/11 DKO cells were still capable of releasing IL-1β, and their IL-1β levels were close to those of WT cells at 24 h (Fig. 2A).

Indeed, MLKL KO cells had reduced processing of caspase-1, IL-1β, and GSDMD at 12 and 24 h postinfection (Fig. 2C). The remaining levels of IL-1β p17 and GSDMD p30 were comparable to those of the caspase-1/11 DKO cells at 12 h, suggesting that IAV-induced inflammasome activation requires MLKL at the early stage (Fig. 2C). In contrast, canonical NLRP3 inflammasome-mediated IL-1β secretion and cell death were dependent on caspase-1/11 but not on RIPK3 or MLKL (Fig. 2D and E). Necroptosis triggered by Pam3CSK4 and the pan-caspase inhibitor Z-VAD-FMK required RIPK3 and MLKL but not caspase-1/11 (Fig. 2F). Together, these results support a model in which MLKL promotes inflammasome activation at the earlier stages of IAV infection.

### Caspase-8 regulates IL-1β production in the absence of MLKL-inflammasomes.

**FIG 3:**
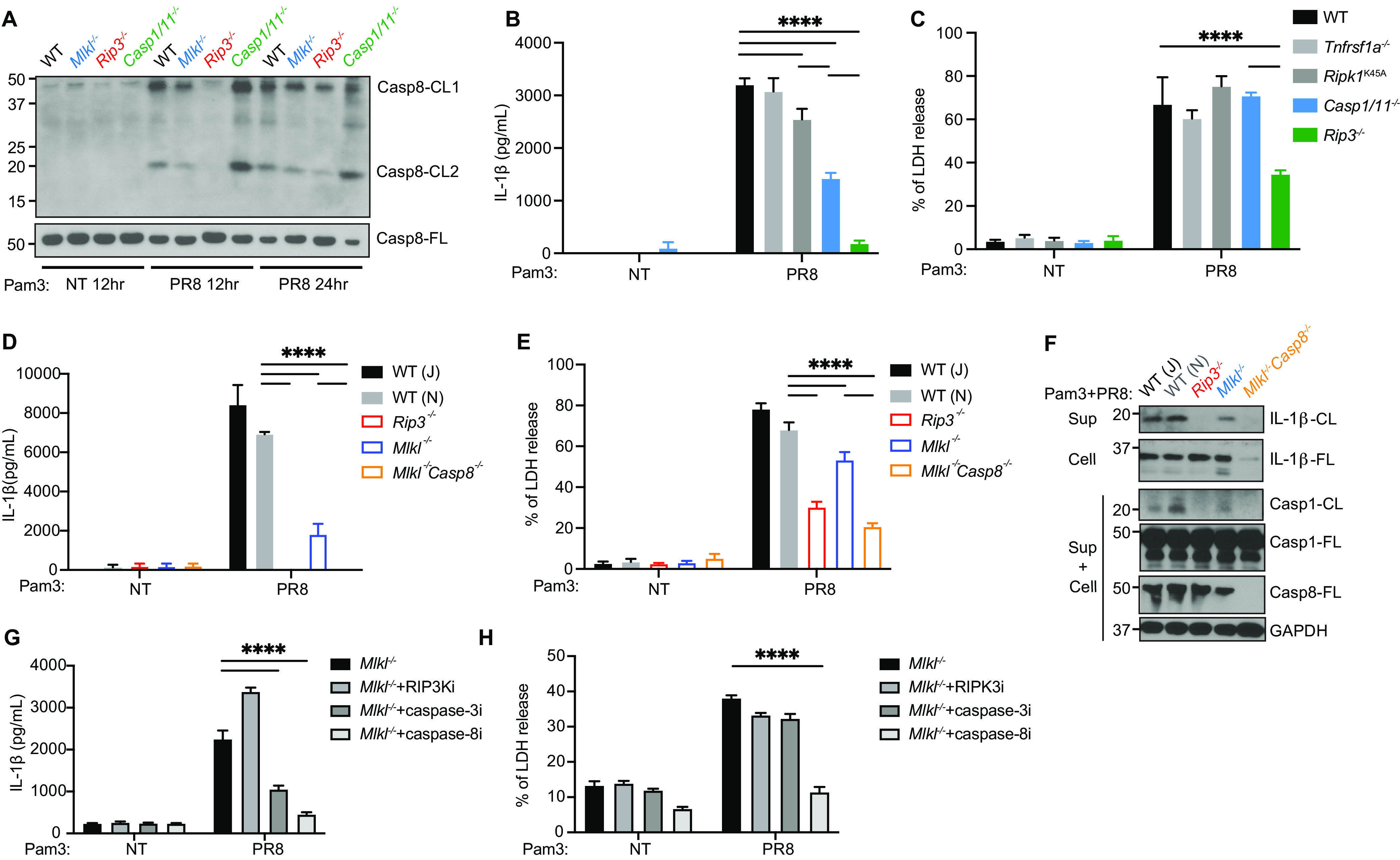
Caspase-8 mediates IL-1β production and cell death in the absence of MLKL-inflammasomes. (A) WT, *Rip3^−/−^*, *Mlkl^−/−^*, and *Casp1/11^−/−^* BMDMs were primed with Pam3 for 3 h. The cells were then infected with PR8 at a MOI of 2 or with no virus (NT). Western blots showing caspase-8 in the composite of cell lysate and supernatant. Full-length (-FL) or cleaved forms (-CL) of the protein were indicated. (B and C) WT, *Tnfrsf1a^−/−^*, *Ripk1^K45A^*, *Casp1/11^−/−^*, and *Rip3^−/−^* BMDMs were primed with Pam3 for 3 h and infected with PR8 at a MOI of 2. The IL-1β (B) and LDH (C) levels in the supernatant were determined 18 h after infection. (D–F) BL6/J WT (J), BL6/N WT (N), *Rip3^−/−^*, *Mlkl^−/−^*, and *Mlkl^−/−^Casp8^−/−^* BMDMs were primed with Pam3 for 3 h and infected with PR8 at a MOI of 2. The IL-1β (panel D) and LDH (panel E) levels in the supernatant were determined 18 h after infection. Western blots (panel F) detecting IL-1β-CL in the supernatant, IL-1β-FL in the cell lysate, and caspase-1, caspase-8, and GAPDH in the composite of cell lysate and supernatant. The full-length (-FL) or cleaved form (-CL) of the protein is indicated. (G and H) *Mlkl^−/−^* BMDMs were primed with Pam3 for 3 h and infected with PR8 at a MOI of 2 or with no virus (NT). 2μM GSK’872 (RIPK3i, 50μM Z-DEVD-FMK (caspase-3i), and 50μM Z-IETD-FMK (caspase-8i) were added 1 h later. The IL-1β (panel G) and LDH (panel H) levels in the supernatant were determined 18 h after infection. The data show one representative experiment of at least two independent experiments and are presented as the mean with the SD. N = 3 (replicate wells). The data were analyzed via a two-way ANOVA. ns, not significant; *, *P* < 0.0332; **, *P* < 0.0021; ***, *P* < 0.0002; ****, *P* < 0.0001.

BMDMs can still process and release IL-1β in the absence of caspase-1/11 and MLKL (Fig. 2A and C). MLKL and caspase-1/11 DKO cells also had reduced but residual levels of the GSDMD p30 fragment (Fig. 2C). Moreover, IAV infection led to a p21 GSDMD product that was not affected by MLKL or caspase-1/11 deficiencies (Fig. 2C). Previous studies have shown that caspase-8 could generate IL-1β p17 and GSDMD p30 pore-forming fragments (15, 16). Further, the downstream apoptotic caspases, caspase-3/7, can cleave GSDMD at position Asp88, resulting in the inactive p43 and p21 fragments (19). Indeed, MLKL KO and caspase-1/11 DKO cells had normal, if not more, caspase-8 cleavage after IAV infection (Fig. 3A). We hypothesized that the caspase-8-mediated cell death pathway acts in parallel with the MLKL-inflammasome axis and contributes to the remaining IL-β production and cell death.

Caspase-8 can be activated through the TLR and TNFR pathways, which also signal to MLKL when caspase-8 is inhibited. In most of these cases, MLKL activation and caspase-8 activation require RIPK1 enzymatic activity (24–26). However, in IAV infection, TNFR1 KO and RIPK1^K45A^ kinase-inactive cells had normal IL-1β production and cell death, supporting the claim that caspase-8 and MLKL downstream of ZBP1-RIPK3 but not TNFR or RIPK1 phosphorylation are involved (Fig. 3B and C).

Deleting both MLKL and caspase-8 further blocked the release of IL-1β, compared to MLKL KO cells, and rescued IAV-induced cell death, phenocopying RIPK3 KO cells (Fig. 3D and E). However, caspase-8 has an essential scaffold function regulating NF-κB signaling (27–30) and is required for the transcriptional upregulation of pro-IL-1β (Fig. 3F). To minimize this effect, we used small molecules inhibiting caspase-8 and caspase-3, respectively, in the background of MLKL deficiency after Pam3CSK4 priming (Fig. 3G and H). Treating MLKL KO cells with the caspase-8 inhibitor Z-IETD-FMK recapitulated the phenotype of the MLKL/caspase-8 DKO cells, in that IL-1β release was abolished and cell death was rescued (Fig. 3G and H). In contrast, the RIPK3 inhibition and caspase-3 inhibitor Z-DEVD-FMK did not affect IAV-induced cell death in MLKL KO cells (Fig. 3H). The inhibition of caspase-3, which is downstream of caspase-8 in the apoptosis pathway, partially reduced the remaining IL-1β levels in MLKL KO cells (Fig. 3G). RIPK3 inhibition did not repress IL-1β in MLKL KO cells, confirming its specificity for MLKL (Fig. 3G). Together, those results support that caspase-8 and MLKL serve as two major pathways for IL-1β production and cell death during IAV infection.

### MLKL-mediated K^+^ efflux drives NLRP3 inflammasome activation.

**FIG 4:**
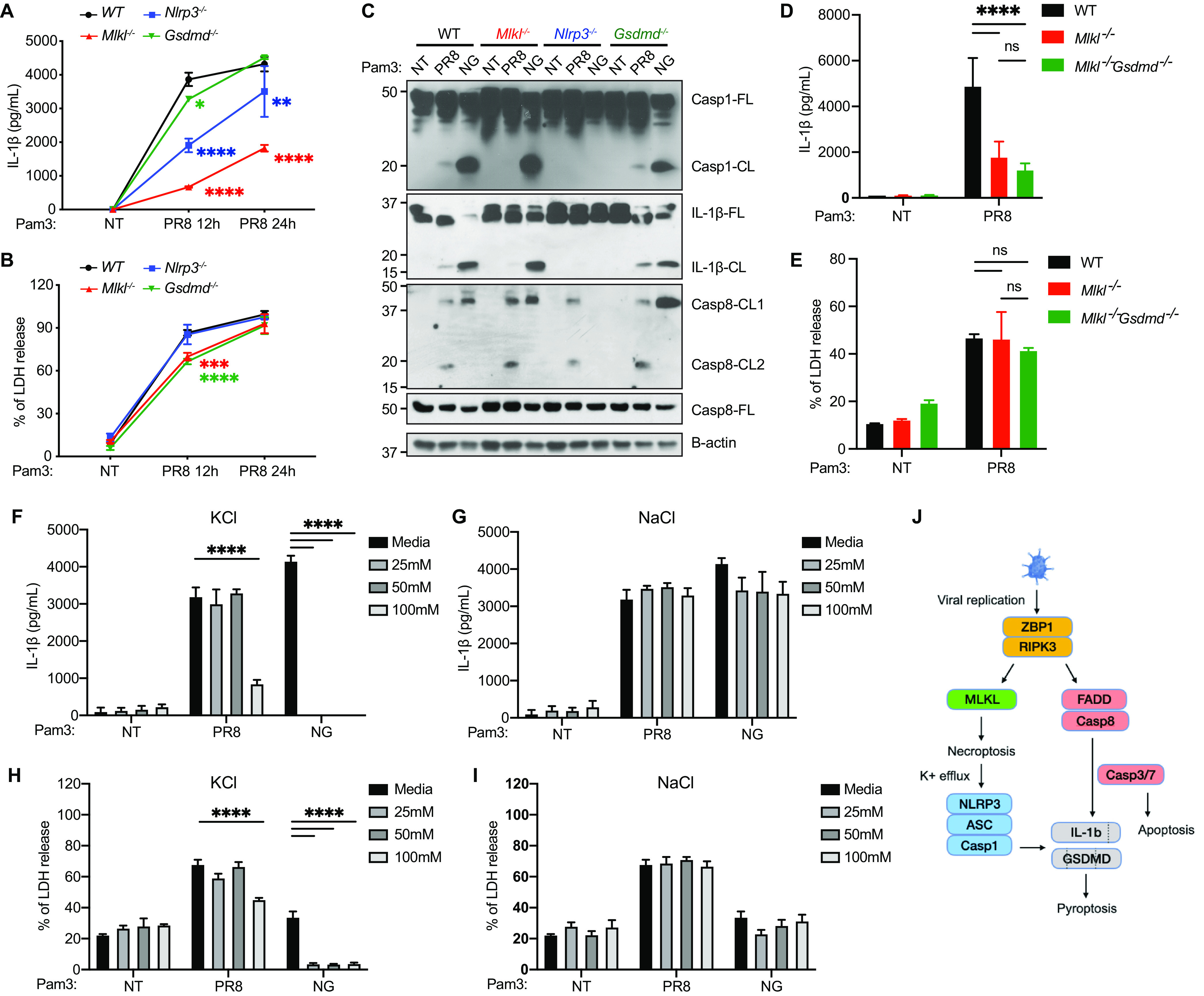
Necroptosis drives inflammasome activation partially through K^+^ efflux and NLRP3. (A and B) WT, *Mlkl^−/−^*, *Nlrp3^−/−^*, and *Gsdmd^−/−^* BMDMs were primed with Pam3 for 3 h. The cells were then infected with PR8 at a MOI of 2 or with no virus (NT). IL-1β (A) and LDH release (B) in the supernatant were determined 12 and 24 h after infection. (C) As described in (panel A), the cells were treated with no virus (NT) and PR8 at a MOI of 2 for 12 h or with nigericin (NG) for 1 h. Caspase-1, IL-1β, caspase-8, and B-actin in the combined cell lysate and the supernatant were detected via Western blotting. The full-length (-FL) or cleaved form (-CL) of the protein is indicated. (D and E) WT, *Mlkl^−/−^*, and *Mlkl^−/−^Gsdmd^−/−^* BMDMs were primed with Pam3 for 3 h. The cells were then infected with PR8 at a MOI of 2 or with no virus (NT). IL-1β (panel D) and LDH release (panel E) in the supernatant were determined 18 h after infection. (F–I) WT BMDMs were primed with Pam3 for 3 h and were then treated with no virus (NT) or with PR8 at a MOI of 2. The indicated concentration of KCl or NaCl was added 1 h postinfection. For the nigericin (NG)-treated cells, KCl or NaCl was added 30 min before the NG stimulations. IL-1β (F and G) and LDH release (H and I) in the supernatant were determined 18 h after the viral infection and 1 h after the NG treatment. (J) Model of IAV-induced inflammatory cell death, based on data from Fig. 1–4. The data show one representative experiment of at least two independent experiments and are presented as the mean with the SD. N = 3 (replicate wells). The data were analyzed via a two-way ANOVA. ns, not significant; *, *P* < 0.0332; **, *P* < 0.0021; ***, *P* < 0.0002; ****, *P* < 0.0001.

IAV infection has been shown to activate the NLRP3 inflammasome (7–9). Given that MLKL-inflammasomes acts in the same axis, we hypothesized that MLKL drives NLRP3 inflammasome activation. We compared IL-1β levels and cell death in WT, MLKL KO, NLRP3 KO, and GSDMD KO cells after IAV infections (Fig. 4A–C). NLRP3 KO cells produced significantly less IL-1β at 12 h; however, the remaining IL-1β level was higher than that observed in the MLKL KO cells (Fig. 4A). These data suggest that NLRP3 partially accounts for the MLKL-mediated inflammasome activation. GSDMD alone, which lies downstream of caspase-1/11 and caspase-8 activation, was dispensable for caspase-1 cleavage, IL-1β cleavage, and IL-1β release (Fig. 4A–C). This suggests that inflammasome activation is not driven by the GSDMD pores that are downstream of caspase-8 and that IL-1β is released through GSDMD-independent mechanisms (Fig. 4C). Together, these observations further support that MLKL acts upstream of inflammasome activation.

Caspase-8 cleavage remained intact in MLKL KO, NLRP3 KO, and GSDMD KO cells, consistent with the fact that those cells had largely normal cell deaths (Fig. 4B and C). Deleting both MLKL and GSDMD did not alter IAV-induced IL-1β release or cell death, compared to MLKL KO cells (Fig. 4D and E), which suggests that additional effectors, other than GSDMD, are required downstream of caspase-8.

One converged trigger for the NLRP3 inflammasome is potassium efflux (31). MLKL pores that execute necroptosis can cause potassium efflux and can activate the NLRP3 inflammasome (20). We investigated whether this mechanism contributes to inflammasome activation in IAV infection and tested it by blocking the ion efflux with extracellular ions (Fig. 4F–I). As expected, extracellular K^+^ but not Na^+^ at as low of a concentration as 25 mM shut down the canonical NLRP3 inflammasome that was activated by nigericin, a K^+^-H^+^ ionophore. IAV-induced inflammasome activation, indicated by the release of IL-1β, was also inhibited by extracellular K^+^, but this only occurred at 100 mM, which is a much higher threshold than that observed with the nigericin-stimulated NLRP3 inflammasome. Although it remains unclear why the thresholds were different, these data suggest that MLKL activates the inflammasome partially through necroptosis-mediated K^+^ efflux, which activates NLRP3.

### MLKL is dispensable for host responses to IAV infection.

**FIG 5:**
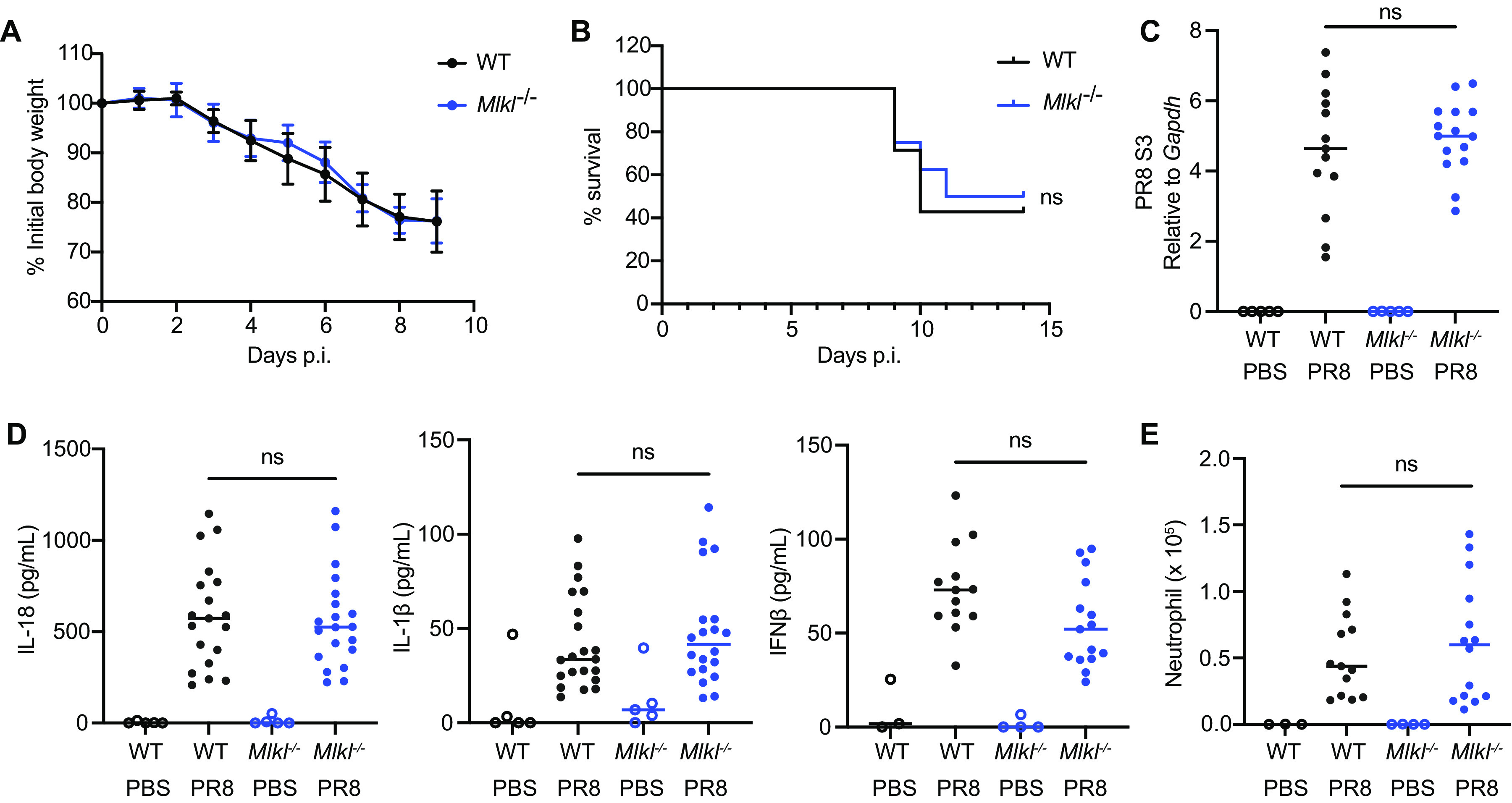
MLKL is dispensable for host responses to IAV infection. (A and B) WT and *Mlkl^−/−^* mice (age- and sex-matched, cohoused) were intranasally infected with PR8 (20 PFU) and monitored for morbidity, daily. Body weight (A) and survival (B) are shown. (C–E) WT and *Mlkl^−/−^* mice (age- and sex-matched, cohoused) were intranasally infected with PR8 (8,000 PFU) for 48 h. The viral RNA in the total lung tissues was determined via qPCR (C). The cytokine levels in the bronchoalveolar lavage fluid were determined via ELISA (D), and the cellularity was analyzed via flow cytometry (E). The data were combined from at least two independent experiments. Each dot represents an individual mouse. The mean with the SD is presented for the body weight data, and the median is presented for the other data. The data were analyzed via a two-way ANOVA. ns, not significant; *, *P* < 0.0332; **, *P* < 0.0021; ***, *P* < 0.0002; ****, *P* < 0.0001.

Next, we investigated the role of MLKL *in vivo* by using a mouse model of IAV infection. We observed that MLKL KO mice were comparable with WT mice in terms of body weight loss and survival at a sublethal dose (Fig. 5A and B). The production of IL-1β and IL-18 as well as the recruitment of neutrophils are all hallmarks of inflammasome-mediated inflammatory responses. To examine these responses, we used a lethal dose of IAV and analyzed the mice 48 h after infection. Similar amounts of viral genome were detected in the lungs of WT and MLKL KO mice (Fig. 5C). The IL-18, IL-1β, and IFN-β levels in the bronchoalveolar lavage fluid from MLKL KO mice were comparable to those of the WT mice (Fig. 5D). The neutrophil numbers were also largely comparable (Fig. 5E). Together, these data support that MLKL serves as a redundant mechanism downstream of RIPK3 and that MLKL alone is dispensable for host inflammatory responses against IAV *in vivo*.

## DISCUSSION

IAV infection leads to multiple forms of cell death, including apoptosis, necroptosis, and pyroptosis, through the common regulator RIPK3. Here, we demonstrated that the three death pathways have a hierarchy and interplay with each other in IAV-infected cells. Apoptosis and necroptosis are the primary pathways downstream of RIPK3. They act redundantly to promote not only cell death but also the processing and release of the proinflammatory cytokine IL-1β. At the earlier stages of infection, MLKL-mediated necroptosis drives inflammasome activation, contributing to the maturation of IL-1β. In the absence of the MLKL-inflammasome axis, the production of IL-1β is delayed. However, active caspase-8 is sufficient for IL-1β processing and secretion at these later stages. In these cases, necroptosis and caspase-8-dependent pathways eventually lead to an inflammatory form of cell death.

Inflammatory cell death is highly immunogenic. It is crucial in the antiviral response in that it recruits neutrophils and monocytes to the site of infection, which activates adjacent immune cells for effector functions and promotes the development of adaptive immune responses (7–9, 32, 33). Additionally, the cell deaths of infected cells can halt viral replication and can the prevent further dissemination of the virus (34). The interplay of the cell death pathways in IAV-infected cells, especially myeloid cells that express components of all three pathways, ensures the immunogenic outcome and viral restriction, even when viruses inhibit parts of the pathways. Indeed, pandemic but not seasonal H1N1 strains blocked cell death in human dendritic cells, suggesting that successful viral evasion correlates with enhanced pathogenicity (35).

RIPK3 KO mice were more susceptible to IAV infections (2, 3, 36, 37), whereas RIPK3 kinase-dead mutant or MLKL KO mice were comparable to WT mice (2, 36). We also observed that MLKL KO mice had comparable survival to WT mice after an IAV infection (Fig. 5). This supports our hypothesis that the redundant caspase-8 pathway is sufficient for driving inflammatory cell death. On the other hand, *Casp8^DA/DA^Mlkl^−/−^* mice but not *Casp8^DA/DA^* mice were more susceptible to IAV infection (38). *Casp8^DA/DA^* mutants had impaired apoptosis without affecting necroptosis and NF-κB signaling. These data support that the MLKL-inflammasome pathway is also sufficient in the absence of apoptosis.

The importance of inflammasome components for IAV infections was first identified *in vivo*. ASC KO, caspase-1/11 DKO, and IL-1R KO mice were more susceptible to IAV infections, whereas NLRP3 KO mice had moderate to normal susceptibility, compared with WT mice (7–9, 32). We also found that NLRP3 KO BMDMs had higher remaining levels of IL-1β, compared to MLKL KO cells, suggesting that NLRP3-independent inflammasomes are also involved (Fig. 4). However, the milder phenotype of the MLKL KO mice, compared to mice that were deficient in inflammasome components, conflicted with the *in vitro* observations that MLKL KO BMDMs had impaired inflammasome activation. One potential reason could be that non-myeloid cells have different mechanisms of activating inflammasomes independently of MLKL and NLRP3. In support of this idea, a recent report showed that MxA, rather than NLRP3, induces ASC oligomerization and caspase-1 activation in respiratory epithelial cells (39).

Taken together, the results of our study provided a novel mechanism for IAV-induced inflammasome activation and dissected the redundant roles of the apoptosis and necroptosis pathways in driving inflammatory cell death.

## MATERIALS AND METHODS

### Mice.

The mice were maintained in a specific pathogen-free (SPF) facility at the University of Massachusetts Chan Medical School. All of the animal experiments were approved by the Institutional Animal Care and Use Committee. The *Rip3^−/−^*, *Mlkl^−/−^*, and *Tnfrsf1a^−/−^* mice were obtained from Michelle Kelliher at UMass Chan Medical School (40–42). The *Rip3^−/−^* and *Gsdmd^−/−^* mice were generated by Vishwa Dixit at Genentech (13). The *Mlkl^−/−^* and *Tnfrsf1a^−/−^* mice were from Jackson Laboratories. The *Mlkl^−/−^Gsdmd^−/−^* and *Mlkl^−/−^Casp8^−/−^* mice were bred in-house. The *Nlrp3^−/−^* and *Pycard^−/−^* mice were kindly provided by John Bertin at Millennium Pharmaceuticals (Takeda). The *Ripk1^K45A^* mutant mice were generated at GlaxoSmithKline and were provided by John Bertin (43). The *Casp1/11^−/−^* (129 crossed to C57BL/6) mice were obtained from Michael N. Starnback (44, 45). The WT C57BL/6J and WT C57BL/6N mice were obtained from Jackson Laboratory and were bred in-house.

The Influenza A/Puerto Rico/8/1934 H1N1 virus (PR8) was grown in SPF embryonated chicken eggs and was purchased from Charles River Laboratories (10100374). The mice were anesthetized using isoflurane and were intranasally inoculated with 25 μL diluted PR8 in PBS or PBS control. The mice were monitored for body weight and disease daily. Alternatively, bronchoalveolar lavage (BAL) fluids and whole lung tissues were collected at 2 days postinfection.

### Cell culture.

Bone marrow cells were harvested from approximately 6 to 24-week-old mice and were removed of red blood cells using red blood cell lysis buffer (Sigma). Cells were cultured in DMEM supplemented with 10% (vol/vol) fetal calf serum (FCS), 1× Penicillin-Streptomycin (Corning), and L929 conditioned media containing M-CSF. Fresh medium was added every 3 days. BMDMs were split and used for experiments after at least 6 days of differentiation.

BMDMs were seeded the day before the stimulations. Cells were primed with 100 ng/mL Pam3CSK4 (Invivogen, tlrl-pms) for approximately 2 to 3 h. For the IAV infections, cells were washed twice with PBS and incubated with PR8 diluted in 0.05% (wt/vol) bovine serum albumin (BSA)/PBS for 2 h at 37°C. X-Vivo 10 media (Lonza, 04-743Q) containing FCS was added to a final concentration of 3% (vol/vol) FCS. Supernatant, cells, or a mixture of supernatant and cells were collected at various time points for downstream analysis.

For the IAV infections with inhibitors, the inhibitors were added after 2 h of viral incubation. For the nigericin (10 μM, Sigma, N7143) treatment, the inhibitors were added after priming and 30 min before the nigericin: RIPK3 inhibitor GSK’872 (Tocris), MLKL and GSDMD inhibitor necrosulfonamide (Cayman), pan-caspase inhibitor Z-VAD-FMK (Santa Cruz), caspase-3 inhibitor Z-DEVD-FMK (Santa Cruz), caspase-8 inhibitor Z-IETD-FMK (R&D).

### Cell death assays.

For real-time live cell imaging to monitor cell death, BMDMs seeded in 96-well plates were incubated with 1 μg/mL Hoechst (ThermoFisher) and 0.25 μΜ Sytox Orange (ThermoFisher) for 30 min. For the IAV infections, dyes were added after 2 h of incubation with the virus. For the nigericin treatment, dyes were added after priming and 30 min before the nigericin was added. The plates were read using a Cytation 5 instrument (BioTek) with the temperature controlled at 37°C and the CO_2_ controlled. The percentage of Sytox Orange-positive cells in the Hoechst positive-cells was calculated using the Gen5 software package.

Alternatively, the percentage of maximum lactate dehydrogenase (LDH) release was determined using a CytoTox 96 Nonradioactive Cytotoxicity Assay (Promega).

### Western blot.

Protein was extracted using RIPA buffer (ThermoFisher). Lysates were added with SDS sample loading buffer and dithiothreitol (DTT). Denatured samples were separated via SDS-PAGE and were transferred to nitrocellulose membranes. The membranes were blocked using fresh 2% nonfat milk in 1× tris buffered saline with Tween 20 (Santa Cruz, sc-362311) at room temperature for 1 h before incubation with antibodies at 4°C overnight. After washing, the membranes were incubated with secondary antibodies at room temperature for 1 h and were visualized using an Odyssey Imaging System (LICOR) or X-ray films. Antibodies: mouse caspase-1 (Adipogen, AG-20-B-0042-C100), mouse GSDMD (Abcam, ab209845), mouse IL-1β (R&D, AF-401-NA), full-length mouse caspase-8 (Cell Signaling Technology, CST, 1C12), cleaved mouse caspase-8 (CST, D5B2), mouse/human β-actin (Sigma, AC-15), mouse/human GAPDH (Sigma, GAPDH-71.1), IRdye 800C goat anti-rabbit IgG (LICOR, 926-32211), IRDye 680RD goat anti-mouse IgG (LICOR, 926-68070), goat anti-rabbit IgG HRP (Bio-Rad, 1705046), goat anti-mouse IgG HRP (Bio-Rad, 1721011).

### ELISA.

The mouse IL-1β and IFN-β levels were determined using DuoSet ELISA Kits (R&D). Reagents for mouse IL-18 ELISA: capture antibody (R&D, D047-3), detection antibody (R&D, D048-5), and recombinant mouse IL-18 standards (R&D, B004-5).

### cDNA synthesis and real-time PCR.

Mouse whole lungs were harvested in QIAzol (Qiagen) and were homogenized. Total RNA was extracted using chloroform, per the manufacturer’s instructions. The RNA was quantified via NanoDrop. 500 ng of RNA were reversed transcribed using an iScript cDNA Synthesis Kit (Bio-Rad). Real-time PCR was performed using SYBR green supermix (Bio-Rad) with detection on a CFX96 system (Bio-Rad). The primers were ordered from Integrated DNA Technologies. The relative expression was determined via 2-dCt, using *Gapdh* as the reference gene.

### Flow cytometry.

Cells were centrifuged from BAL fluids and were resuspended in FACS buffer (PBS, 5% FCS, 2 mM EDTA, and 0.05% sodium azide). Cells were stained for the viability dye and surface antigens on ice for 30 min. After washing with FACS buffer, the cells were fixed on ice for 15 min using 1% paraformaldehyde (PFA). Fixed cells were supplemented with PKH25 reference microbeads (Sigma) and were analyzed on a Cytek Aurora spectral cytometer. The flow data were analyzed using FlowJo. Antibodies and dyes: CD45.2 (104), CD11B (M1/70), CD11C (N418), Ly6G (1A8), Ly6C (HK1.1), MHC-II (M5/114.15.2), F4/80 (BM8), Ghost Dye Violet 540 (Tonbo, 13-0879).

## References

[1] Kuriakose T, Man SM, Malireddi RK, Karki R, Kesavardhana S, Place DE, Neale G, Vogel P, Kanneganti TD. 2016. ZBP1/DAI is an innate sensor of influenza virus triggering the NLRP3 inflammasome and programmed cell death pathways. Sci Immunol 1. doi:10.1126/sciimmunol.aag2045. PMC5131924 27917412

[2] Nogusa S, Thapa RJ, Dillon CP, Liedmann S, Oguin TH, 3rd, Ingram JP, Rodriguez DA, Kosoff R, Sharma S, Sturm O, Verbist K, Gough PJ, Bertin J, Hartmann BM, Sealfon SC, Kaiser WJ, Mocarski ES, Lopez CB, Thomas PG, Oberst A, Green DR, Balachandran S. 2016. RIPK3 activates parallel pathways of MLKL-driven necroptosis and FADD-mediated apoptosis to protect against influenza A virus. Cell Host Microbe 20:13–24. doi:10.1016/j.chom.2016.05.011. 27321907 PMC5026823

[3] Zhang T, Yin C, Boyd DF, Quarato G, Ingram JP, Shubina M, Ragan KB, Ishizuka T, Crawford JC, Tummers B, Rodriguez DA, Xue J, Peri S, Kaiser WJ, Lopez CB, Xu Y, Upton JW, Thomas PG, Green DR, Balachandran S. 2020. Influenza virus Z-RNAs induce ZBP1-mediated necroptosis. Cell 180:1115–1129. doi:10.1016/j.cell.2020.02.050. 32200799 PMC7153753

[4] Sun L, Wang H, Wang Z, He S, Chen S, Liao D, Wang L, Yan J, Liu W, Lei X, Wang X. 2012. Mixed lineage kinase domain-like protein mediates necrosis signaling downstream of RIP3 kinase. Cell 148:213–227. doi:10.1016/j.cell.2011.11.031. 22265413

[5] Wang H, Sun L, Su L, Rizo J, Liu L, Wang LF, Wang FS, Wang X. 2014. Mixed lineage kinase domain-like protein MLKL causes necrotic membrane disruption upon phosphorylation by RIP3. Mol Cell 54:133–146. doi:10.1016/j.molcel.2014.03.003. 24703947

[6] Zhao C, Zhao W. 2020. NLRP3 inflammasome-a key player in antiviral responses. Front Immunol 11:211. doi:10.3389/fimmu.2020.00211. 32133002 PMC7040071

[7] Allen IC, Scull MA, Moore CB, Holl EK, McElvania-TeKippe E, Taxman DJ, Guthrie EH, Pickles RJ, Ting JP. 2009. The NLRP3 inflammasome mediates in vivo innate immunity to influenza A virus through recognition of viral RNA. Immunity 30:556–565. doi:10.1016/j.immuni.2009.02.005. 19362020 PMC2803103

[8] Ichinohe T, Lee HK, Ogura Y, Flavell R, Iwasaki A. 2009. Inflammasome recognition of influenza virus is essential for adaptive immune responses. J Exp Med 206:79–87. doi:10.1084/jem.20081667. 19139171 PMC2626661

[9] Thomas PG, Dash P, Aldridge JR, Jr., Ellebedy AH, Reynolds C, Funk AJ, Martin WJ, Lamkanfi M, Webby RJ, Boyd KL, Doherty PC, Kanneganti TD. 2009. The intracellular sensor NLRP3 mediates key innate and healing responses to influenza A virus via the regulation of caspase-1. Immunity 30:566–575. doi:10.1016/j.immuni.2009.02.006. 19362023 PMC2765464

[10] Bauernfeind FG, Horvath G, Stutz A, Alnemri ES, MacDonald K, Speert D, Fernandes-Alnemri T, Wu J, Monks BG, Fitzgerald KA, Hornung V, Latz E. 2009. Cutting edge: NF-kappaB activating pattern recognition and cytokine receptors license NLRP3 inflammasome activation by regulating NLRP3 expression. J Immunol 183:787–791. doi:10.4049/jimmunol.0901363. 19570822 PMC2824855

[11] Juliana C, Fernandes-Alnemri T, Kang S, Farias A, Qin F, Alnemri ES. 2012. Non-transcriptional priming and deubiquitination regulate NLRP3 inflammasome activation. J Biol Chem 287:36617–36622. doi:10.1074/jbc.M112.407130. 22948162 PMC3476327

[12] Martinon F, Burns K, Tschopp J. 2002. The inflammasome: a molecular platform triggering activation of inflammatory caspases and processing of proIL-beta. Mol Cell 10:417–426. doi:10.1016/S1097-2765(02)00599-3. 12191486

[13] Kayagaki N, Stowe IB, Lee BL, O'Rourke K, Anderson K, Warming S, Cuellar T, Haley B, Roose-Girma M, Phung QT, Liu PS, Lill JR, Li H, Wu J, Kummerfeld S, Zhang J, Lee WP, Snipas SJ, Salvesen GS, Morris LX, Fitzgerald L, Zhang Y, Bertram EM, Goodnow CC, Dixit VM. 2015. Caspase-11 cleaves gasdermin D for non-canonical inflammasome signalling. Nature 526:666–671. doi:10.1038/nature15541. 26375259

[14] Shi J, Zhao Y, Wang K, Shi X, Wang Y, Huang H, Zhuang Y, Cai T, Wang F, Shao F. 2015. Cleavage of GSDMD by inflammatory caspases determines pyroptotic cell death. Nature 526:660–665. doi:10.1038/nature15514. 26375003

[15] Orning P, Weng D, Starheim K, Ratner D, Best Z, Lee B, Brooks A, Xia S, Wu H, Kelliher MA, Berger SB, Gough PJ, Bertin J, Proulx MM, Goguen JD, Kayagaki N, Fitzgerald KA, Lien E. 2018. Pathogen blockade of TAK1 triggers caspase-8-dependent cleavage of gasdermin D and cell death. Science 362:1064–1069. doi:10.1126/science.aau2818. 30361383 PMC6522129

[16] Maelfait J, Vercammen E, Janssens S, Schotte P, Haegman M, Magez S, Beyaert R. 2008. Stimulation of Toll-like receptor 3 and 4 induces interleukin-1beta maturation by caspase-8. J Exp Med 205:1967–1973. doi:10.1084/jem.20071632. 18725521 PMC2526192

[17] Gringhuis SI, Kaptein TM, Wevers BA, Theelen B, van der Vlist M, Boekhout T, Geijtenbeek TB. 2012. Dectin-1 is an extracellular pathogen sensor for the induction and processing of IL-1beta via a noncanonical caspase-8 inflammasome. Nat Immunol 13:246–254. doi:10.1038/ni.2222. 22267217

[18] Ruhl S, Broz P. 2015. Caspase-11 activates a canonical NLRP3 inflammasome by promoting K(+) efflux. Eur J Immunol 45:2927–2936. doi:10.1002/eji.201545772. 26173909

[19] Taabazuing CY, Okondo MC, Bachovchin DA. 2017. Pyroptosis and apoptosis pathways engage in bidirectional crosstalk in monocytes and macrophages. Cell Chem Biol 24:507–514. doi:10.1016/j.chembiol.2017.03.009. 28392147 PMC5467448

[20] Conos SA, Chen KW, De Nardo D, Hara H, Whitehead L, Nunez G, Masters SL, Murphy JM, Schroder K, Vaux DL, Lawlor KE, Lindqvist LM, Vince JE. 2017. Active MLKL triggers the NLRP3 inflammasome in a cell-intrinsic manner. Proc Natl Acad Sci USA 114:E961–E969. 28096356 10.1073/pnas.1613305114 PMC5307433

[21] Ichinohe T, Pang IK, Iwasaki A. 2010. Influenza virus activates inflammasomes via its intracellular M2 ion channel. Nat Immunol 11:404–410. doi:10.1038/ni.1861. 20383149 PMC2857582

[22] Rodriguez AE, Bogart C, Gilbert CM, McCullers JA, Smith AM, Kanneganti TD, Lupfer CR. 2019. Enhanced IL-1β production is mediated by a TLR2-MYD88-NLRP3 signaling axis during coinfection with influenza A virus and Streptococcus pneumoniae. PLoS One 14:e0212236. doi:10.1371/journal.pone.0212236. 30794604 PMC6386446

[23] Rathkey JK, Zhao J, Liu Z, Chen Y, Yang J, Kondolf HC, Benson BL, Chirieleison SM, Huang AY, Dubyak GR, Xiao TS, Li X, Abbott DW. 2018. Chemical disruption of the pyroptotic pore-forming protein gasdermin D inhibits inflammatory cell death and sepsis. Sci Immunol 3. doi:10.1126/sciimmunol.aat2738. PMC6462819 30143556

[24] Holler N, Zaru R, Micheau O, Thome M, Attinger A, Valitutti S, Bodmer JL, Schneider P, Seed B, Tschopp J. 2000. Fas triggers an alternative, caspase-8-independent cell death pathway using the kinase RIP as effector molecule. Nat Immunol 1:489–495. doi:10.1038/82732. 11101870

[25] Wang L, Du F, Wang X. 2008. TNF-alpha induces two distinct caspase-8 activation pathways. Cell 133:693–703. doi:10.1016/j.cell.2008.03.036. 18485876

[26] Humphries F, Yang S, Wang B, Moynagh PN. 2015. RIP kinases: key decision makers in cell death and innate immunity. Cell Death Differ 22:225–236. doi:10.1038/cdd.2014.126. 25146926 PMC4291486

[27] Weng D, Marty-Roix R, Ganesan S, Proulx MK, Vladimer GI, Kaiser WJ, Mocarski ES, Pouliot K, Chan FK, Kelliher MA, Harris PA, Bertin J, Gough PJ, Shayakhmetov DM, Goguen JD, Fitzgerald KA, Silverman N, Lien E. 2014. Caspase-8 and RIP kinases regulate bacteria-induced innate immune responses and cell death. Proc Natl Acad Sci USA 111:7391–7396. doi:10.1073/pnas.1403477111. 24799678 PMC4034196

[28] Philip NH, DeLaney A, Peterson LW, Santos-Marrero M, Grier JT, Sun Y, Wynosky-Dolfi MA, Zwack EE, Hu B, Olsen TM, Rongvaux A, Pope SD, Lopez CB, Oberst A, Beiting DP, Henao-Mejia J, Brodsky IE. 2016. Activity of uncleaved caspase-8 controls anti-bacterial immune defense and TLR-induced cytokine production independent of cell death. PLoS Pathog 12:e1005910. doi:10.1371/journal.ppat.1005910. 27737018 PMC5063320

[29] Chaudhary PM, Eby MT, Jasmin A, Kumar A, Liu L, Hood L. 2000. Activation of the NF-kappaB pathway by caspase 8 and its homologs. Oncogene 19:4451–4460. doi:10.1038/sj.onc.1203812. 11002417

[30] Henry CM, Martin SJ. 2017. Caspase-8 acts in a non-enzymatic role as a scaffold for assembly of a pro-inflammatory “FADDosome” complex upon TRAIL stimulation. Mol Cell 65:715–729. doi:10.1016/j.molcel.2017.01.022. 28212752

[31] Yang Y, Wang H, Kouadir M, Song H, Shi F. 2019. Recent advances in the mechanisms of NLRP3 inflammasome activation and its inhibitors. Cell Death Dis 10:128. doi:10.1038/s41419-019-1413-8. 30755589 PMC6372664

[32] Schmitz N, Kurrer M, Bachmann MF, Kopf M. 2005. Interleukin-1 is responsible for acute lung immunopathology but increases survival of respiratory influenza virus infection. J Virol 79:6441–6448. doi:10.1128/JVI.79.10.6441-6448.2005. 15858027 PMC1091664

[33] Pang IK, Ichinohe T, Iwasaki A. 2013. IL-1R signaling in dendritic cells replaces pattern-recognition receptors in promoting CD8(+) T cell responses to influenza A virus. Nat Immunol 14:246–253. doi:10.1038/ni.2514. 23314004 PMC3577947

[34] Meischel T, Villalon-Letelier F, Saunders PM, Reading PC, Londrigan SL. 2020. Influenza A virus interactions with macrophages: lessons from epithelial cells. Cell Microbiol 22:e13170. doi:10.1111/cmi.13170. 31990121

[35] Hartmann BM, Albrecht RA, Zaslavsky E, Nudelman G, Pincas H, Marjanovic N, Schotsaert M, Martinez-Romero C, Fenutria R, Ingram JP, Ramos I, Fernandez-Sesma A, Balachandran S, Garcia-Sastre A, Sealfon SC. 2017. Pandemic H1N1 influenza A viruses suppress immunogenic RIPK3-driven dendritic cell death. Nat Commun 8:1931. doi:10.1038/s41467-017-02035-9. 29203926 PMC5715119

[36] Oltean T, Van San E, Divert T, Vanden Berghe T, Saelens X, Maelfait J, Takahashi N, Vandenabeele P. 2021. Viral dosing of influenza A infection reveals involvement of RIPK3 and FADD, but not MLKL. Cell Death Dis 12:471. doi:10.1038/s41419-021-03746-0. 33976111 PMC8113499

[37] Downey J, Pernet E, Coulombe F, Allard B, Meunier I, Jaworska J, Qureshi S, Vinh DC, Martin JG, Joubert P, Divangahi M. 2017. RIPK3 interacts with MAVS to regulate type I IFN-mediated immunity to Influenza A virus infection. PLoS Pathog 13:e1006326. doi:10.1371/journal.ppat.1006326. 28410401 PMC5406035

[38] Shubina M, Tummers B, Boyd DF, Zhang T, Yin C, Gautam A, Guo XJ, Rodriguez DA, Kaiser WJ, Vogel P, Green DR, Thomas PG, Balachandran S. 2020. Necroptosis restricts influenza A virus as a stand-alone cell death mechanism. J Exp Med 217. doi:10.1084/jem.20191259. PMC7596817 32797196

[39] Lee S, Ishitsuka A, Noguchi M, Hirohama M, Fujiyasu Y, Petric PP, Schwemmle M, Staeheli P, Nagata K, Kawaguchi A. 2019. Influenza restriction factor MxA functions as inflammasome sensor in the respiratory epithelium. Sci Immunol 4. doi:10.1126/sciimmunol.aau4643. 31653718

[40] Murphy JM, Czabotar PE, Hildebrand JM, Lucet IS, Zhang JG, Alvarez-Diaz S, Lewis R, Lalaoui N, Metcalf D, Webb AI, Young SN, Varghese LN, Tannahill GM, Hatchell EC, Majewski IJ, Okamoto T, Dobson RC, Hilton DJ, Babon JJ, Nicola NA, Strasser A, Silke J, Alexander WS. 2013. The pseudokinase MLKL mediates necroptosis via a molecular switch mechanism. Immunity 39:443–453. doi:10.1016/j.immuni.2013.06.018. 24012422

[41] Newton K, Sun X, Dixit VM. 2004. Kinase RIP3 is dispensable for normal NF-kappa Bs, signaling by the B-cell and T-cell receptors, tumor necrosis factor receptor 1, and Toll-like receptors 2 and 4. Mol Cell Biol 24:1464–1469. doi:10.1128/MCB.24.4.1464-1469.2004. 14749364 PMC344190

[42] Peschon JJ, Torrance DS, Stocking KL, Glaccum MB, Otten C, Willis CR, Charrier K, Morrissey PJ, Ware CB, Mohler KM. 1998. TNF receptor-deficient mice reveal divergent roles for p55 and p75 in several models of inflammation. J Immunol 160:943–952. doi:10.4049/jimmunol.160.2.943. 9551933

[43] Berger SB, Kasparcova V, Hoffman S, Swift B, Dare L, Schaeffer M, Capriotti C, Cook M, Finger J, Hughes-Earle A, Harris PA, Kaiser WJ, Mocarski ES, Bertin J, Gough PJ. 2014. Cutting Edge: RIP1 kinase activity is dispensable for normal development but is a key regulator of inflammation in SHARPIN-deficient mice. J Immunol 192:5476–5480. doi:10.4049/jimmunol.1400499. 24821972 PMC4048763

[44] Li P, Allen H, Banerjee S, Franklin S, Herzog L, Johnston C, McDowell J, Paskind M, Rodman L, Salfeld J. 1995. Mice deficient in IL-1 beta-converting enzyme are defective in production of mature IL-1 beta and resistant to endotoxic shock. Cell 80:401–411. doi:10.1016/0092-8674(95)90490-5. 7859282

[45] van der Velden AW, Velasquez M, Starnbach MN. 2003. Salmonella rapidly kill dendritic cells via a caspase-1-dependent mechanism. J Immunol 171:6742–6749. doi:10.4049/jimmunol.171.12.6742. 14662878

