# Supplementary figures and images for: MLKL-Driven Inflammasome Activation and Caspase-8 Mediate Inflammatory Cell Death in Influenza A Virus Infection
Source: mBio. 2023 Feb 28;14(2):e00110-23. doi: 10.1128/mbio.00110-23 (PMC10127685; doi:10.1128/mbio.00110-23)

**Figure S1**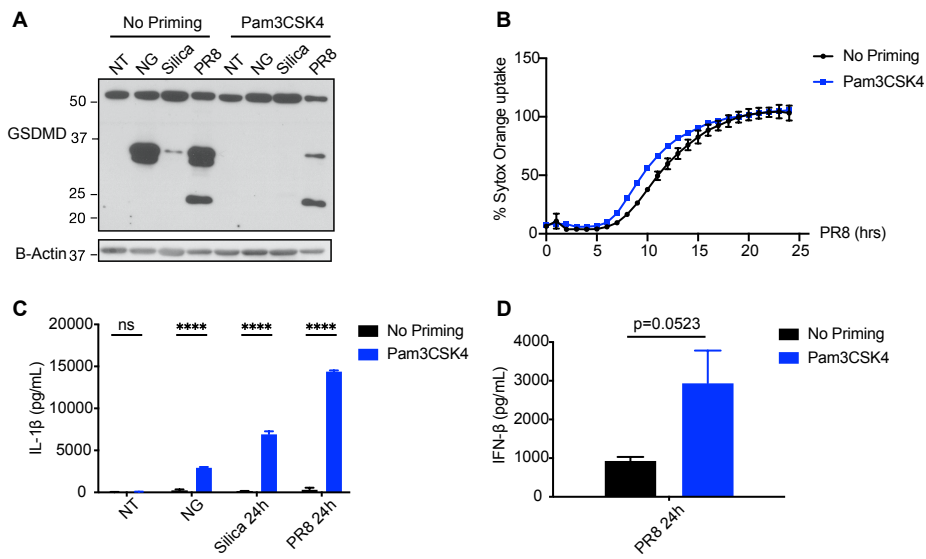

Supplement: FIG S1 [file mbio.00110-23-s0001.pdf]
